# Metabarcoding Analysis Reveals Microbial Diversity and Its Environmental Drivers in the Pantanos de Villa Lagoons in Lima—Peru

**DOI:** 10.1002/ece3.73039

**Published:** 2026-03-12

**Authors:** Camila Castillo‐Vilcahuaman, Roger Alberto Palomino Huarcaya, Pedro Sepúlveda‐Rebolledo, Maribel Baylon Cortitoma

**Affiliations:** ^1^ Facultad de Ciencia e Ingeniería Universidad Peruana Cayetano Heredia Lima Peru; ^2^ Laboratorio de Microbiología y Biotecnología Microbiana Universidad Nacional Mayor de San Marcos Lima Peru; ^3^ CCTE Ciencia y Vida Santiago Chile; ^4^ Laboratorio de Ecología Molecular y Biodiversidad, Facultad de Ciencias Biológicas Universidad Nacional Mayor de San Marcos Lima Peru

**Keywords:** coastal wetlands, environmental drivers, metabarcoding, microbial communities

## Abstract

Coastal wetlands are ecosystems of ecological importance that face multiple threats from contamination. While their ecological value for local flora and fauna is well known, the microbial diversity of this habitat has not been characterized before. This study characterized, for the first time, the microbial diversity in four lagoons of the Pantanos de Villa Wildlife Refuge using the 16S rRNA marker gene metabarcoding and identified the physicochemical drivers that influence the microbial community structure of these lagoons. Nine sampling sites were established distributed across the Delicias, Génesis, Marvilla, and Mayor lagoons, conducting three seasonal samplings (May, August, and October). Physicochemical parameters were measured in each sampled lagoon (temperature, electrical conductivity, pH, dissolved oxygen, total dissolved solids, and salinity). The results revealed that the most identified phylum at the bacterial level was Pseudomonadota, followed by Bacteroidota. At the archaeal level, the abundance of Nanoarchaeota was observed in all sampled lagoons, followed by Lokiarchaeota. The Delicias lagoon, previously classified as the most contaminated lagoon in the wetland by previous studies, showed parameters such as high electrical conductivity, total dissolved solids, and salinity when compared to the other lagoons, which was reflected in a distinctive microbial community structure strongly associated with these parameters. On the other hand, the Marvilla lagoon, closer to the coast, showed a microbial community strongly influenced by its more alkalinic pH. This study constitutes the first determination of microbial diversity in Pantanos de Villa and demonstrates how local environmental conditions—natural or human‐induced—shape microbial diversity. These findings provide baseline information for future monitoring of this protected coastal wetland and highlight the importance of environmental factors such as pH and electrical conductivity that determine the structure of microbial communities in these coastal wetlands.

## Graphical abstract

This study characterized, for the first time, the microbial diversity in four lagoons of the Pantanos de Villa Wildlife Refuge in Lima, Peru, using 16S rRNA marker gene metabarcoding and identified the physicochemical drivers that influence the microbial community structure of these lagoons.

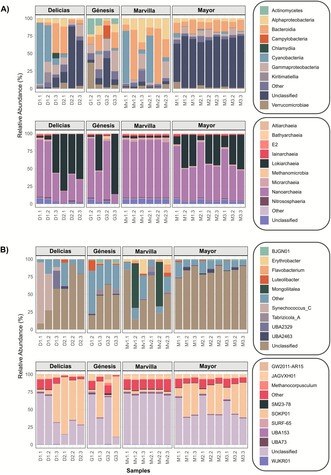

## 1 Introduction

Coastal wetlands are highly productive transitional ecosystems that provide benefits such as water purification, flood control, carbon sequestration, and biodiversity conservation (Xu et al. 2019). However, these habitats face multiple challenges such as urbanization, water usage, mining, and pollution. These anthropogenic processes alter their ecological structure and function (Newton et al. 2020). Climate change has intensified these threats through sea level rise, water acidification, and extreme weather events (Wang and Gu 2021).

The Pantanos de Villa Wildlife Refuge, located in the south of Lima, constitutes the only coastal wetland with protected natural area status in Peru's capital and has been internationally recognized as a Ramsar site for its high biodiversity and ecosystem services. Their ecological value is considered to be “very good” in relation to other coastal wetlands in Lima and the nearby Callao province (Jurado et al. 2024). This refuge, which covers more than 260 ha, harbors multiple water bodies that support diverse wildlife, including microbial communities (Iannacone and Alvariño 2007). These habitats face constant threats from uncontrolled urban growth, industrial activities, and waste accumulation. This can be seen in the elevated levels of nutrients and thermotolerant coliforms in some lagoons, which are common indicators of anthropogenic contamination (Huaman‐Vilca et al. 2020). These conditions can directly affect aquatic biodiversity, including the microbial communities native to these lagoons.

Microbial diversity has been recognized as a key component of the functioning of lotic ecosystems such as rivers and streams. Microorganisms are involved in ecological processes such as nutrient cycling, organic matter decomposition, and resilience to environmental disturbances (Li et al. 2021). Some studies have been able to prove that microbial composition responds dynamically to environmental variations such as temperature, organic matter quality, and nutrient availability (Zeglin 2015). However, the microbial communities at the Pantanos de Villa Wildlife Refuge remain uncharacterized, and it is still unknown how the environmental conditions found in these wetlands affect their composition.

Metabarcoding, a technique based on high‐throughput sequencing of the 16S rRNA gene, has become an established tool for characterizing microbial communities, overcoming the limitations of classical culture‐based methods (Šubrtová Salmonová and Bunesova 2017). This technique has been used in various aquatic environments, revealing both the taxonomic structure and the spatial and temporal dynamics of communities, and how these are influenced by environmental variables such as pH, conductivity, temperature, nutrients, and dissolved oxygen (Pinar‐Méndez et al. 2022; Wang et al. 2021). Over the last decade, decreased sequencing costs and greater accessibility to bioinformatics tools have enabled more researchers to incorporate 16S metabarcoding into their studies (Weinroth et al. 2022). Given these advantages, 16S rRNA metabarcoding represents an ideal approach for characterizing microbial communities in underexplored ecosystems.

These tools have also proven important for understanding the mechanisms by which bacterial communities respond to environmental drivers. Ballesteros et al. (2023), described the status of microbial communities in seven wetlands in Bogotá, Colombia with different anthropogenic influences. For this, they used 16S and 18S metabarcoding, with which they were able to observe how the structure of these communities was affected by human interventions (Ballesteros et al. 2023).

This study aims to characterize the microbial diversity in four lagoons located in the Pantanos de Villa Wildlife Refuge (Delicias, Génesis, Marvilla, and Mayor) using 16S metabarcoding, and to identify the physicochemical parameters that influence the structure of their communities. The microbial community in this coastal wetland was characterized through a seasonal sampling and the effect of environmental variables such as pH, conductivity, temperature, dissolved oxygen, and salinity on community composition was evaluated. This research represents the first detailed characterization of bacterial and archaeal diversity in Pantanos de Villa and offers a baseline for monitoring these ecosystems in the face of the increasing anthropogenic influence on this refuge. Understanding how environmental factors shape microbial communities allows us to anticipate early ecological changes and design more effective conservation strategies, recognizing microorganisms as sensitive indicators of the overall health of the ecosystem.

## 2 Methods

### 2.1 Sampling

**FIGURE 1:**
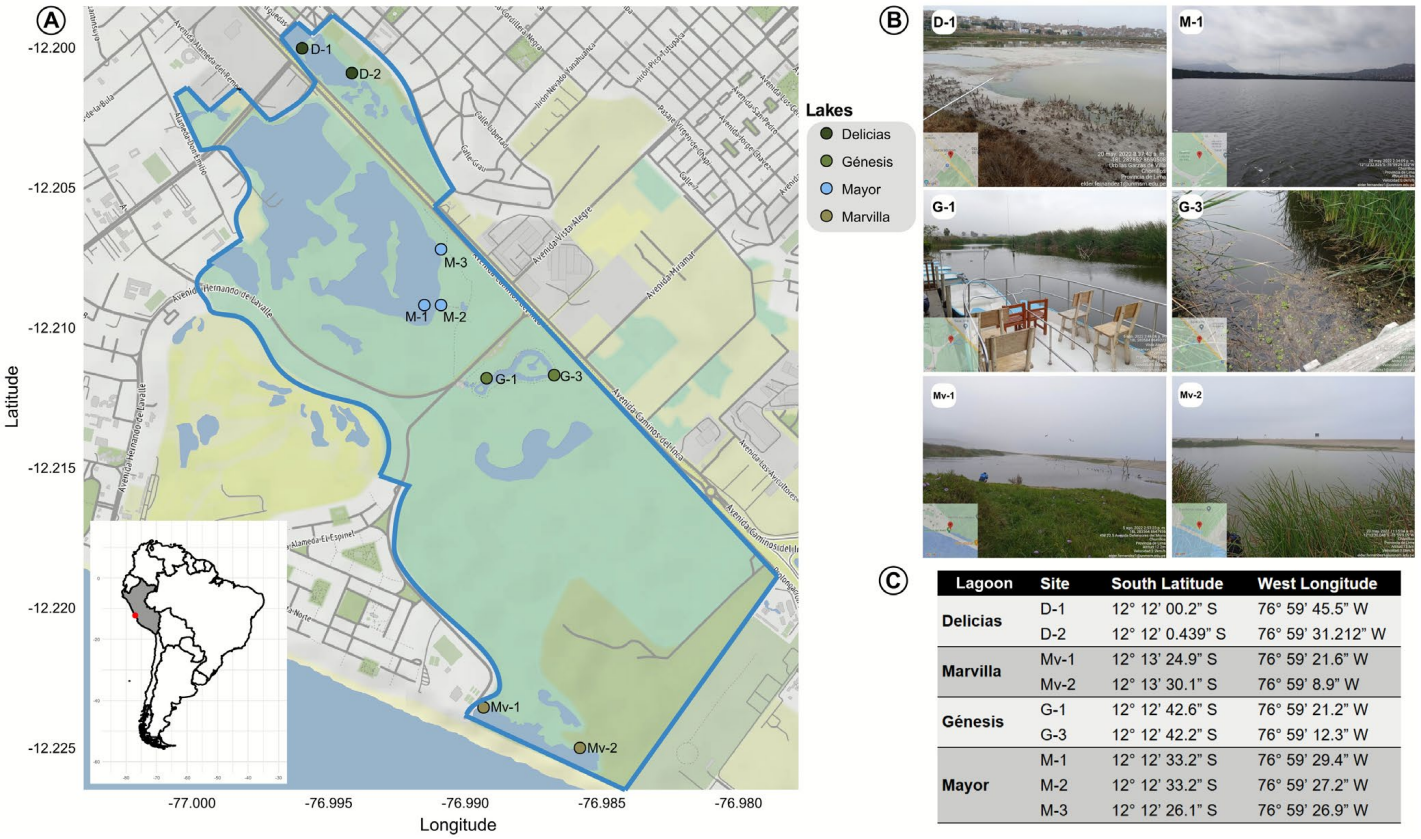
(A) Sampling sites in the Pantanos de Villa area in Lima, Peru, delimited by the blue contour. (B) Photos of some sampling sites. (C) Coordinates of the sampling points.

The study was conducted in the Pantanos de Villa Wildlife Refuge located in the Chorrillos district, in the city of Lima, with the respective collection permits requested from the local authority regulating the natural refuge, PROHVILLA. Nine sampling sites were established considering the lagoons that comprise the refuge (Figure 1). The sampling sites were reached by foot. Because of its size, sampling sites at the Mayor lagoon were reached by boat. Considering the hydrological conditions of this wetland, three samplings were conducted per year on a seasonal basis (May, August, and October). The sampling dates were the May 30, 2022, August 16, 2022, and the October 18, 2022. The study area was drawn using the Maps library v. 3.4.2.1 (Becker et al. 2025), and ggmap v. 4 (Kahle et al. 2025) using the stadiamaps repositories. Map data copyrighted OpenStreetMap contributors and available from https://www.openstreetmap.org.

The following water physicochemical parameters were taken in situ at each sampling site at the surface level: temperature (TE), electrical conductivity (EC), pH, total dissolved solids (TDS), and salinity (SAL). This was done using the Thermo Scientific Orion multiparameter device. For the dissolved oxygen (DO) parameter, a Sper Scientific Direct oximeter was used. For the molecular characterization, 500 mL of surface water was collected using a 2.5 m length sampler in sterile bottles and filtered using a portable pump through a 0.22 μm Sterivex filter, then packed in a refrigerated cooler. These filters were kept refrigerated during the sampling period and subsequently frozen until DNA extraction was performed. Water samples arrived at the laboratory after 2 h of transport.

### 2.2 DNA Extraction and 16S rRNA Sequencing

The total DNA was isolated using a Sterivex filter and the MN NucleoMag DNA/RNA kit for aqueous samples following the manufacturer's instructions (DNA Extraction Protocol). The extraction was carried out in the Laboratory of Molecular Systematics and Phylogeography in the Faculty of Biological Sciences at the Universidad Nacional Mayor de San Marcos. Amplification, library construction, and sequencing of the samples were performed via a commercial service (Sangon Biotech) that used the MR‐DNA technique. The 16S rRNA gene for bacteria was sequenced as a molecular marker, using an Illumina platform (MiSeq). The sequenced 16S rRNA fragment corresponds to the variable V4 region. The 515F (GTGYCAGCMGCCGCGGTAA) and 806R (GGACTACNVGGGTWTCTAAT) universal primers were used for amplification purposes.

### 2.3 Quality Control of Sequences and Taxonomic Assignment

Raw sequences were processed using the DADA2 package v. 1.34.0 (Callahan et al. 2016) in R v. 4.4.2. Quality filtering was performed using a maximum expected error threshold of 2 for both forward and reverse reads and a minimum sequence length requirement of 175 bp. Sequences were trimmed to a length of 225 bp for forward reads and 210 bp for reverse reads. The treated sequences were grouped in Amplicon Sequence Variants (ASVs), which allows a better clustering resolution. Chimeric sequences were removed using the removeBimeraDenovo from the DADA2 package. A variance stabilizing transformation was applied using DESeq2 v. 1.46.0 (Love et al. 2014). Taxonomic assignment was carried out using the taxaAssign algorithm in the DADA2 package, along with the GTDB database v. 4.5 modified for DADA2 (Ali 2024), using a standard configuration. For the decontamination, blanks were used that passed through all the extraction and sequencing procedures. For removing contaminants, the decontam library v. 1.26.0 was used (Davis et al. 2018).

### 2.4 Physicochemical Parameters Analysis

To characterize and compare the environmental conditions across the sampled lagoons, statistical analysis of the obtained physicochemical variables in the lagoons was performed in R. The Kruskal‐Wallis test was used for non‐parametric values due to the low number of samples. To verify the significance of this test, a Dunn test with Bonferroni correction was performed. The Kruskal‐Wallis test was applied using the kruskal.test function while the Dunn test with Bonferroni correction was performed using the Dunn Test library v. 1.3.6 (Dinno 2024). The figures were elaborated using ggplot2 v. 3.5.2 (Wickham et al. 2025) while the significance asterisks and brackets were plotted using the ggpubr library v. 0.6.0 (Kassambara 2025).

### 2.5 Composition and Diversity Analysis

The composition bar charts of the evaluated microbial communities were made using ggplot2. Relative abundances were calculated considering the unknown occurrences in the evaluated samples. Alpha and beta diversity analyses were performed using the phyloseq package v. 1.48.0 (McMurdie and Holmes 2013) in R. Four alpha diversity indices were calculated: observed richness, Shannon index, Simpson index, and inverse Simpson index (using the standard vegan transformation). Differences in alpha diversity between lagoons were evaluated using an ANOVA test followed by Tukey's post hoc test. For beta diversity analysis, two distances were used: Jaccard and Bray‐Curtis. The Jaccard distance was visualized using a cladogram, while the Bray‐Curtis distance was represented in an NMDS plot. The relationship between the identified microbial communities and environmental variables was evaluated using a distance‐based redundancy analysis applied to the previously evaluated Bray‐Curtis distances. This was performed using the vegan package v. 2.6.10 (Oksanen et al. 2025). Visualizations were also implemented using ggplot2.

### 2.6 Correspondence Analysis

To determine the genera significantly associated with the tested ecological variables, a Pearson correlation was performed. This correlation was carried out by normalizing the obtained data with the CLR method using the microbiome package v. 1.26.0 (Lahti and Shetty 2017) in R. The correlation matrix was performed using the Hmisc package v. 5.2.3 (Harrell and Dupont 2025). *p*‐values were adjusted for multiple comparisons using the Benjamini‐Hochberg method to control false discovery (FDR). Correlations with adjusted *p*‐values lower than 0.05 were considered significant. The heatmap representing the associations was created using ggplot2.

## 3 Results

### 3.1 Sequencing Results

A total of 22 samples were sequenced, yielding between 326,018 and 607,824 raw reads per sample. After quality filtering, between 75,470 and 532,177 reads were retained per sample, representing an average retention rate of 85.4%. The DADA2 pipeline identified between 417 and 3783 raw ASVs per sample. Following decontamination to remove potential contaminants, the final dataset contained between 230 and 3808 ASVs per sample (Table 1).

**TABLE 1:** Summary of sequencing depth and ASV recovery per sample. Raw and filtered read counts are shown alongside ASV numbers before and after contaminant removal using the decontam package. Quality filtering was performed using DADA2 with truncation lengths of 225 bp (forward) and 210 bp (reverse).

Sample	Raw reads	Filtered reads	Raw ASVs	ASVs after decontamination
D1.1	406,658	399,549	492	464
D1.2	287,209	275,247	716	657
D1.3	211,471	183,587	1185	1093
D2.1	204,787	69,991	1078	926
D2.2	315,703	274,720	1183	1053
D2.3	238,433	145,577	1600	1461
G1.2	247,197	172,172	1017	881
G1.3	227,182	148,357	1042	910
G3.2	241,988	213,368	3783	3632
G3.3	323,364	206,969	1796	1641
M1.1	334,092	262,914	1162	962
M1.2	224,161	187,618	844	662
M1.3	265,668	230,990	781	608
M2.1	82,069	68,112	417	291
M2.2	194,309	154,965	766	588
M2.3	287,687	245,275	908	715
M3.1	298,702	257,202	1251	1047
M3.2	219,168	184,786	929	733
M3.3	302,768	259,077	978	781
Mv1.1	339,443	335,311	560	501
Mv1.2	279,592	273,227	1152	1049
Mv1.3	143,763	130,404	729	654
Mv2.1	308,107	296,498	857	776
Mv2.2	293,044	289,182	833	759
Mv2.3	212,875	152,874	839	748

### 3.2 Physicochemical Characteristics of the Sampled Lagoons

**FIGURE 2:**
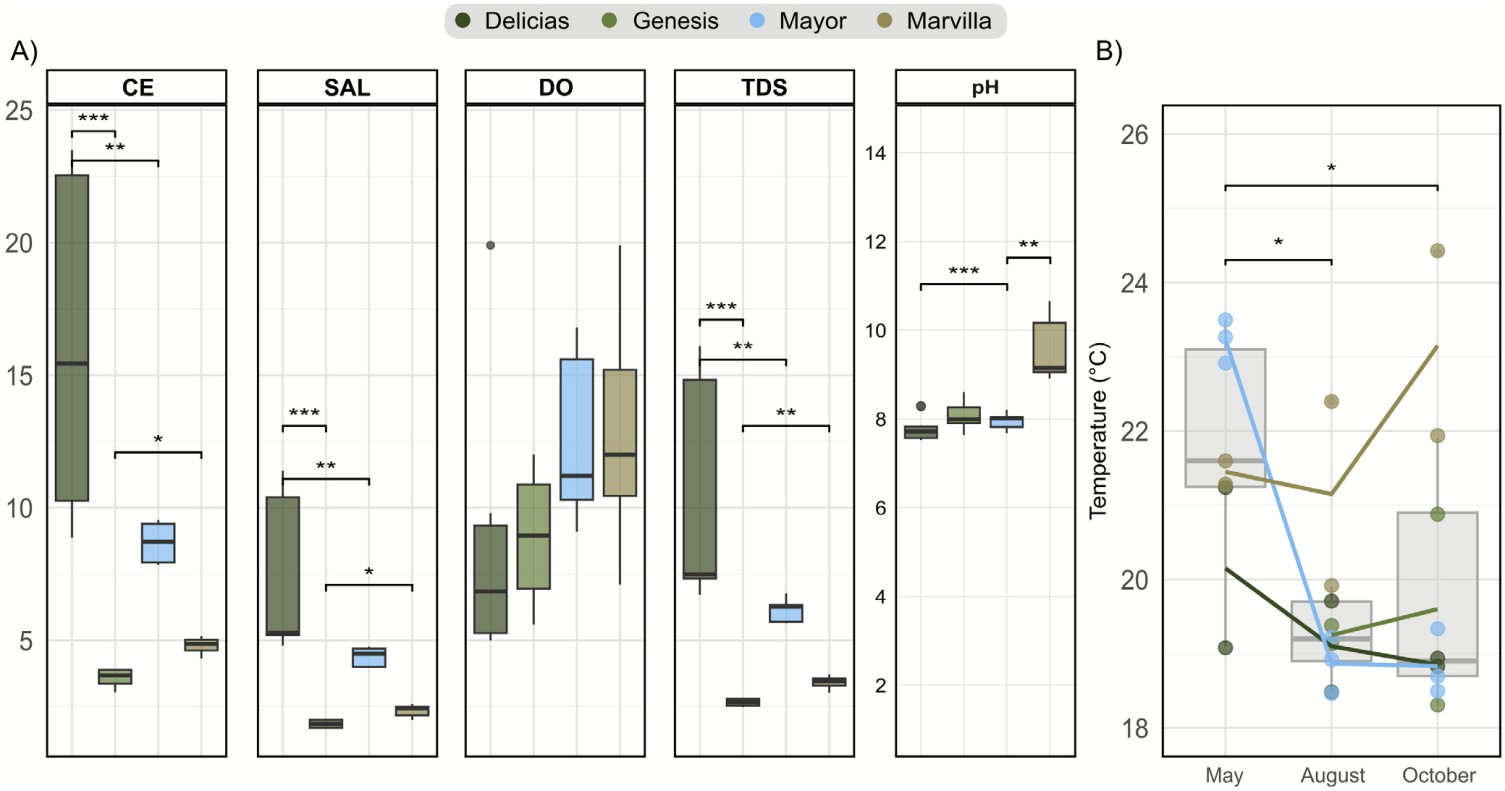
(A) Box‐whisker plot distribution of the obtained physicochemical parameters (temperature (TE), electrical conductivity (EC), pH, dissolved oxygen (DO), total dissolved solids (TDS) and salinity (SAL)) evaluated during sampling. The Delicias lagoon is the one that presents the most significant differences when compared with other lagoons. (B) Distribution of temperature measurements for each evaluated month in addition to representation of mean temperatures. *p < 0.05, **p < 0.01, ***p < 0.001.

The analysis of the parameters measured during sampling in the four lagoons (temperature (TE), electrical conductivity (EC), pH, dissolved oxygen (DO), total dissolved solids (TDS), and salinity (SAL)) revealed that there is variability among them (Figure 2A). In three of the parameters taken in this study, the Delicias lagoon shows a significant difference with respect to Génesis and Mayor lagoons (EC: *p* < 0.01, TDS: *p* < 0.01, and SAL: *p* < 0.01). In the case of the pH measurement, the Delicias lagoon only shows a significant difference with the Mayor lagoon (*p* < 0.001). The only parameter that did not differ significantly between lagoons was DO, where all lagoons presented similar values except for one sample from Delicias lagoon (D2.1). This sample reached an oxygen percentage of 19.9% and a pH value of 8.29 (Table 2).

**TABLE 2:** Physicochemical data obtained at each of the sampling points describe the conditions of each of the samples.

Sample	Lagoon	Month	TE	CE	pH	DO	TDS	SAL
M1.1	Mayor	May	23.5	9.49	7.73	16.7	5.88	4.76
M2.1	Mayor	May	22.9	9.54	7.82	16.8	6.77	4.77
M3.1	Mayor	May	23.3	9.39	7.68	15.6	6.65	4.69
D1.1	Delicias	May	19.1	23.49	7.86	7.9	NaN	NaN
D2.1	Delicias	May	21.2	10.57	7.52	19.9	7.49	5.29
Mv1.1	Marvilla	May	21.6	4.81	9.17	19.9	3.41	2.4
Mv2.1	Marvilla	May	21.3	4.92	8.91	10.4	3.5	2.47
M1.2	Mayor	August	18.9	8.72	7.99	9.1	6.31	4.5
M2.2	Mayor	August	18.5	8.77	8.04	10.6	6.34	4.5
M3.2	Mayor	August	19.2	8.7	8.21	11.2	6.27	4.4
G1.2	Génesis	August	19.1	3.88	8.61	12	2.79	2
G3.2	Génesis	August	19.4	3.91	7.64	5.6	2.81	2
D1.2	Delicias	August	18.5	23.28	8.29	5	16.1	11.4
D2.2	Delicias	August	19.7	10.16	7.74	9.8	7.33	5.2
Mv1.2	Marvilla	August	22.4	5.06	10.5	13.4	3.58	2.5
Mv2.2	Marvilla	August	19.9	5.16	10.66	15.8	3.72	2.6
M1.3	Mayor	October	18.7	7.94	8.04	11.5	5.7	4
M2.3	Mayor	October	18.5	7.87	8.02	10.3	5.66	4
M3.3	Mayor	October	19.3	7.85	8.05	10	5.65	4
G1.3	Génesis	October	18.3	3.05	8.35	10.5	2.56	1.7
G3.3	Génesis	October	20.9	3.48	7.99	7.4	2.49	1.7
D1.3	Delicias	October	18.9	20.3	7.69	5.1	14.82	10.4
D2.3	Delicias	October	18.8	8.88	7.53	5.8	6.72	4.8
Mv1.3	Marvilla	October	24.4	4.31	9.13	10.6	3.03	2.1
Mv2.3	Marvilla	October	21.9	4.56	9.03	7.1	3.26	2

For temperature, a monthly comparison of the mean temperature across lagoons was performed to identify potential seasonal variation (Figure 2B). May exhibits a significant temperature differentiation compared to the other months (*p* < 0.05). It can also be noted that only the Marvilla lagoon exhibited a temperature increase between August and October, although this increase was not statistically significant.

### 3.3 Taxonomic Characterization

**FIGURE 3:**
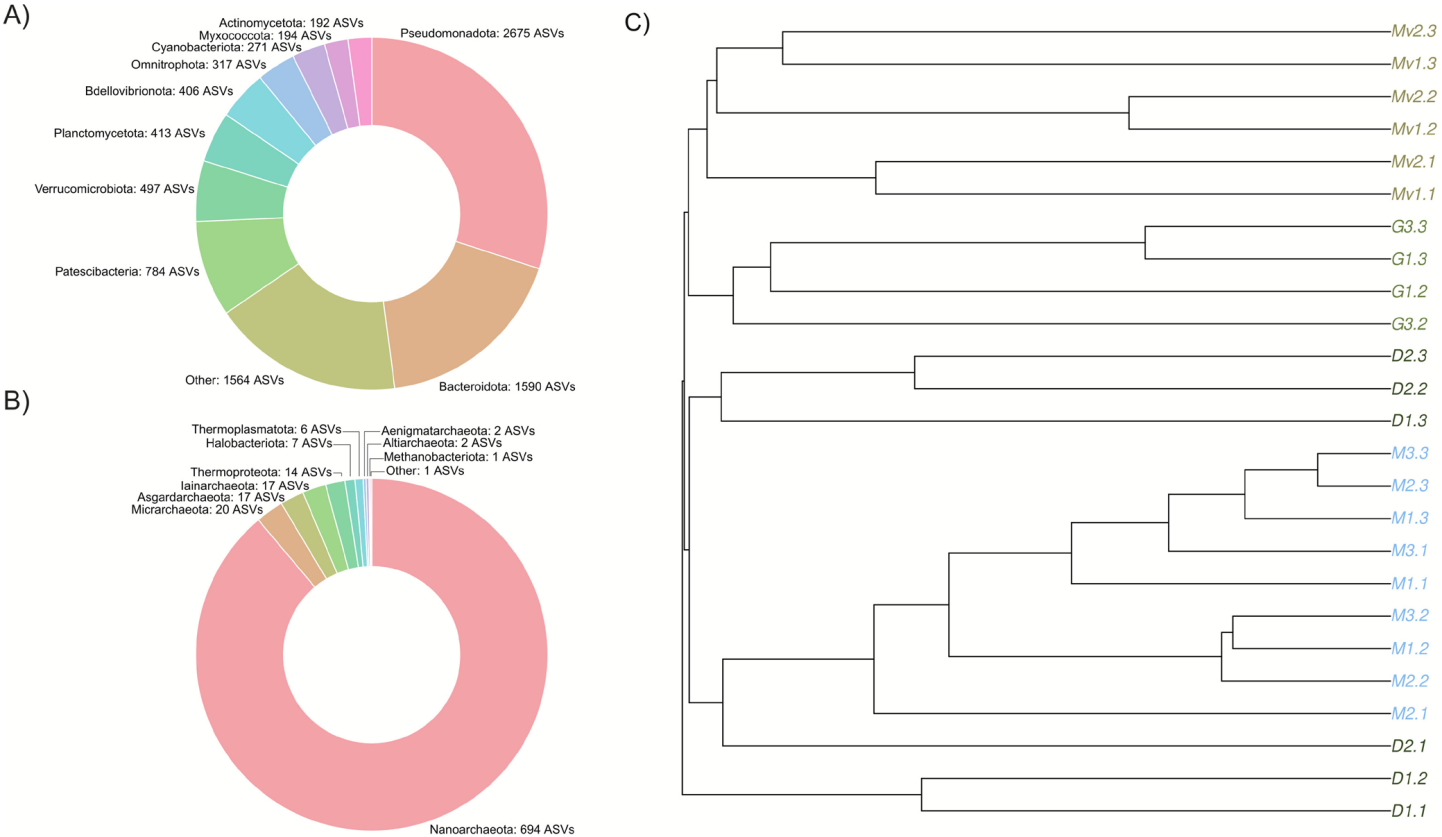
Data initial characterization obtained using 16S. (A, B) show the most frequently identified phyla in unique ASVs recovered from the Bacteria (A) and Archaea (B) domains. Unclassified ASVs were not included. (C) Cladogram created using the Jaccard distance, showing the grouping of samples by lake, except in the case of the Delicias lagoon.

Based on the taxonomic profiling, a count of the unique ASVs found was carried out based on their phylum (Figure 3A). In it, Pseudomonadota turned out to be the phylum with the most incidences in the obtained samples with 2675 unique ASVs. It is followed by the phylum Bacteroidota with 1590 unique ASVs identified in this taxonomic group. Patescibacteria would be the third phylum with 785 unique ASVs identified as such in all samples. 1896 ASVs were not identified in any phylum and were not included in this analysis.

For the case of Figure 3B, all the archaeal phyla found in the samples are described, with the phylum Nanoarchaeota being dominant in this case, showing 694 ASVs grouped here. The next most abundantly identified phylum among the archaeal phyla is Micrarchaeota with 20 ASVs in this group. This phylum is followed by Asgardarchaeota with 17 ASVs identified as such.

Figure 3C shows a cladogram in which the samples from the Marvilla, Mayor, and Génesis lagoons are appropriately grouped according to their geographic location. However, the Delicias lagoon presents three separate clades: one of them is a clade directly related to the clade from the Mayor lagoon, containing samples D2.3, D2.2, and D1.3. There is another branch, containing sample D2.1, which is external to the group belonging to the samples from the Mayor lagoon. Finally, there is a clade that is external to these, composed of samples D1.2 and D1.1. These are even located externally to the rest of the clades containing the samples.

**FIGURE 4:**
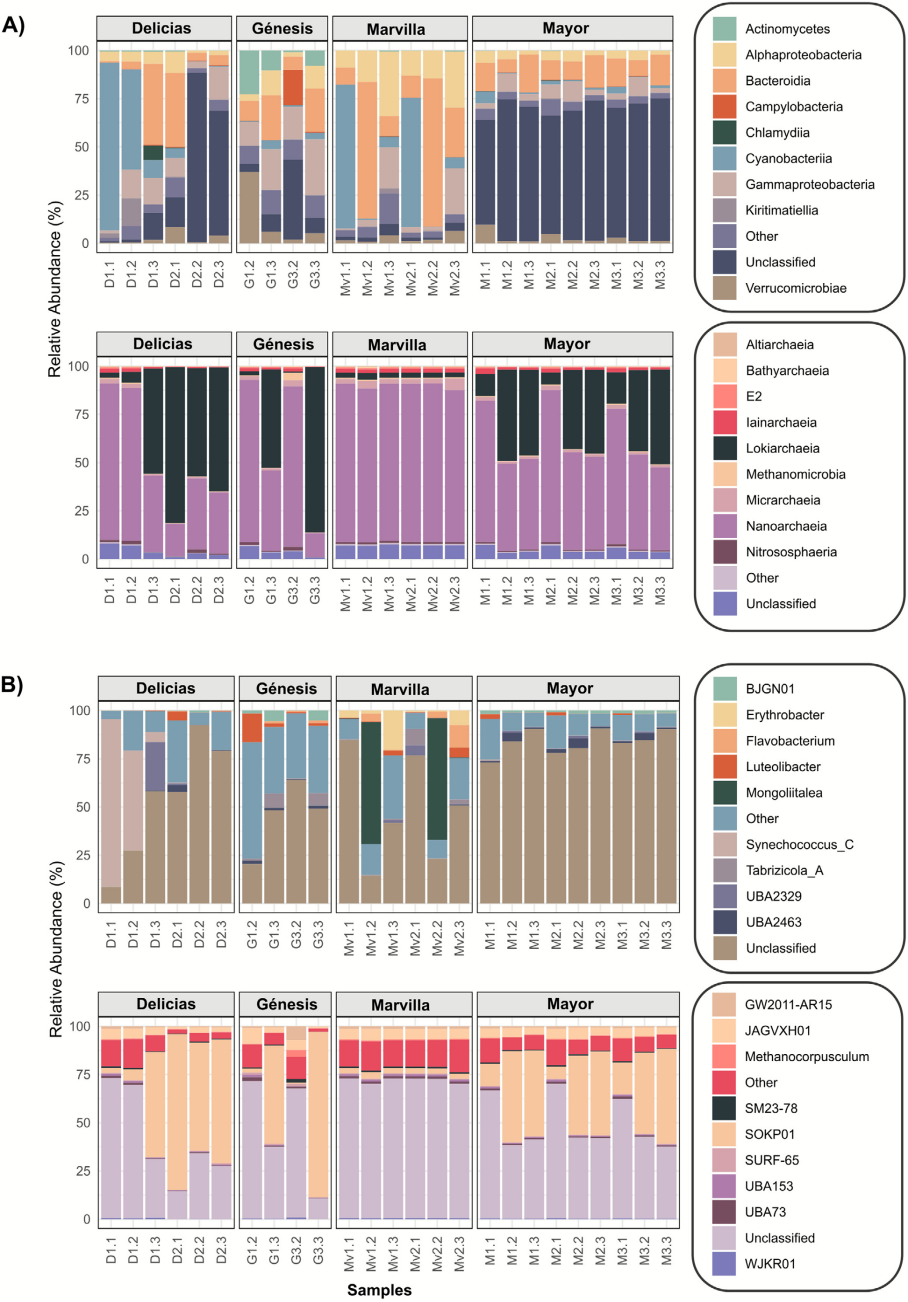
Stacked bars showing the relative abundance of the top 10 taxa present in the sampled lagoons. (A) Bacterial (top) and archaeal (bottom) classes. (B) Bacterial (top) and archaeal (bottom) genera.

Microbial composition analysis at the four study sites revealed distinct taxon diversity patterns for both bacterial and archaeal communities. Figure 4A shows the relative abundance of different bacterial and archaeal classes in Delicias, Génesis, Marvilla, and Mayor. These communities displayed a heterogeneous composition across sampling sites, with a notable predominance of unclassified classes in the Mayor lagoon. In contrast, Delicias and Génesis presented a significant presence of Actinomycetes, Bacteroidia, Gammaproteobacteria, and Cyanobacteria. Furthermore, Marvilla hosts a large proportion of ASVs classified as Cyanobacteria, Bacteroidia, and Alphaproteobacteria, with a notably low proportion of unclassified classes. Regarding the archaeal communities, a predominance of Lokiarchaeia and Nanoarchaeia was observed across all four lagoons, with a similar composition among them.

At the genus level (Figure 4B), distinct patterns were observed across lagoons. In Delicias, *Synechococcus_C* was prevalent at two sampling points. *Luteolibacter* appeared in Delicias during the first month of sampling and in Génesis during the second month. In Marvilla, an abundance of *Mongoliitalea* was observed during the second month of sampling. At the archaeal level, most genera remain and do not have a formal taxonomic designation yet.

Temporal patterns in community composition varied among lagoons. Marvilla exhibited a periodic pattern, in which samples collected during the same month showed similar community composition. In this lagoon, the first month showed a prevalence of the class Cyanobacteria, while the second month was characterized by a prevalence of Bacteroidia. Notably, the genus *Mongoliitalea*, classified within Bacteroidia in this database but now recognized as belonging to the class Cythophagia (Jiang et al. 2022), appeared during this second sampling month. In Mayor, the class Lokiarchaeia was more prevalent during the second and third months of sampling compared to the first. In contrast, neither Delicias nor Génesis exhibited a seasonal pattern.

### 3.4 Diversity of the Microbial Communities

**FIGURE 5:**
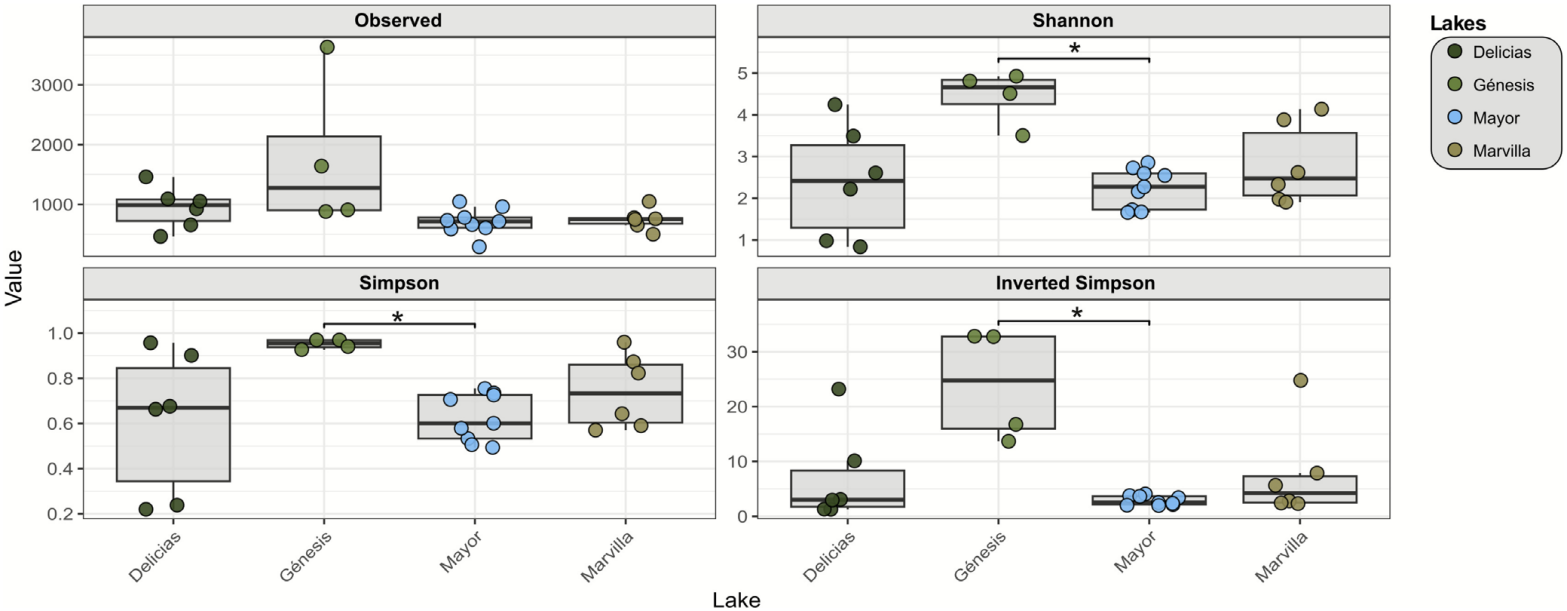
Alpha diversity using different indices. Observed richness and alpha diversity measured with the Shannon, Simpson, and inverted Simpson indices. *p < 0.05.

The observed ASVs richness shows that the Genesis lagoon has samples with a much higher ASV richness than the rest of the lagoons. In the case of the samples evaluated, it is sample G3.2 that stands out in this graph (*n* = 3632). In the case of the rest of the lagoons, the richness obtained was very similar and compact among themselves, showing that only the richness in the Génesis lagoon displayed a greater distribution. Alpha diversity by lagoon was also obtained using 3 indices: Shannon, Simpson, and Inverted Simpson. For all 3 indices, a significant difference was shown between the Génesis and Mayor lagoons (*p* < 0.05), with the Mayor lagoon being the one with the lowest distribution in any of the alpha indices used (Figure 5).

**FIGURE 6:**
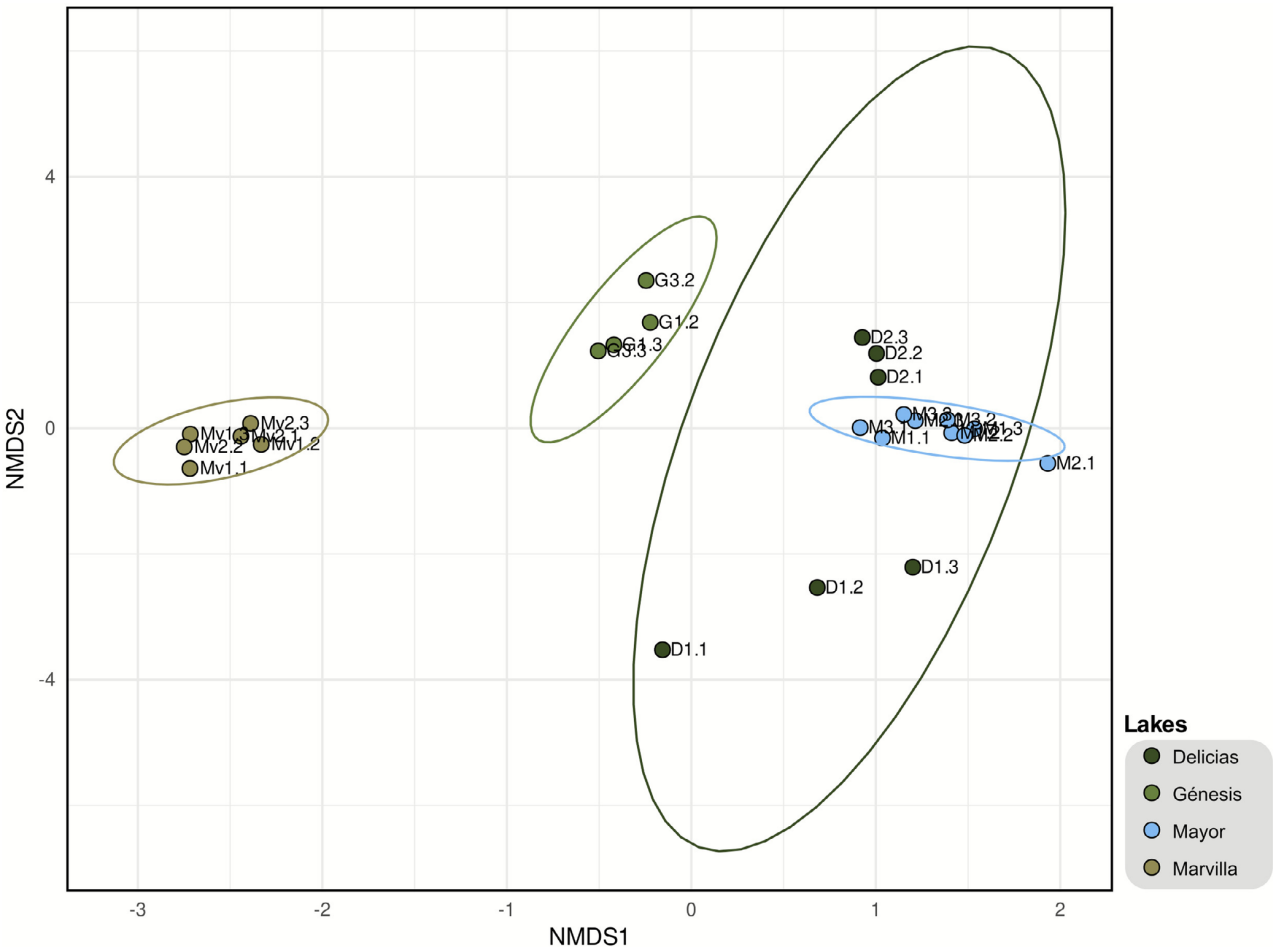
Beta diversity using the Bray‐Curtis index. It is framed within the different clusters found in ellipses.

Regarding beta diversity using the Bray‐Curtis distance (Figure 6), the non‐metric multidimensional scaling (NMDS) ordination analysis clearly clusters the samples according to their site of origin in the case of the Marvilla, Génesis, and Mayor communities. Meanwhile, the microbial community of Delicias is more dispersed. Interestingly, in this analysis, the samples obtained from the sampling site D1 cluster in one site, while the samples from the sampling site D2 cluster in another. Notably, the samples from Marvilla remain on one side of the horizontal axis, while the remaining samples cluster on the opposite side.

### 3.5 Conductivity and pH as Factors Influencing the Microbial Communities in Pantanos de Villa

**FIGURE 7:**
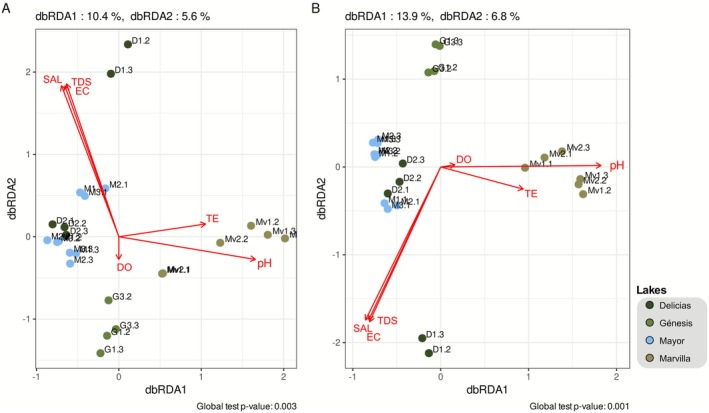
Analysis of the influence of environmental parameters on (A) archaeal and (B) bacterial populations using dbRDA and the Bray‐Curtis distance. D1.1 does not appear in this graph due to the empty values for TDS and SAL.

The influence of environmental variables on the composition of bacterial and archaeal communities was evaluated using distance‐based redundancy analysis (db‐RDA). In the case of archaeal populations (Figure 7A), the obtained model was significant (*p* = 0.001), explaining 10.4% and 5.6% of the variance in the dbRDA1 and dbRDA2 axes, respectively. Among the variables evaluated, pH (*p* = 0.009), conductivity (*p* = 0.036), and temperature (*p* = 0.012) were found to be the main factors influencing archaeal populations. In the case of these populations, the percentage of oxygen, salinity, and dissolved solids did not significantly influence the archaeal structure of the community.

For bacterial populations (Figure 7B), the model was also significant (*p* = 0.003), with 13.9% and 6.8% of the variance explained by the principal axes. Temperature (*p* = 0.007), pH (*p* = 0.001), and conductivity (*p* = 0.001) were the most significant environmental variables. Although the percentage of oxygen did not reach a significant value, an effect was observed outside of this parameter on community structure (*p* = 0.069). Dissolved solids and salinity did not influence the evaluated community.

**FIGURE 8:**
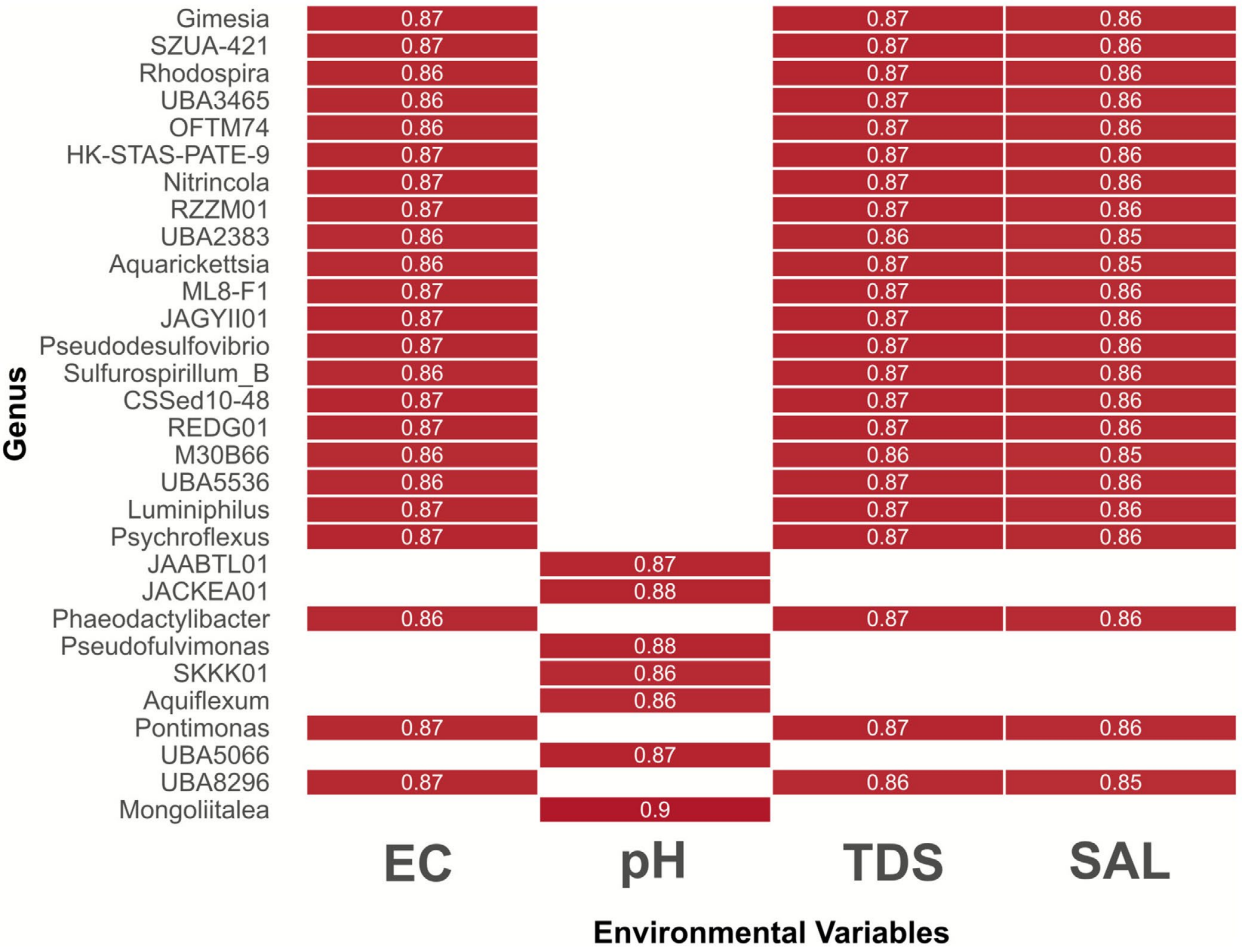
Heatmap showing the Pearson correlation between different genera of bacteria and archaea and environmental factors. Only significant values with an adjusted *p*‐value lower than 0.05 are shown.

Pearson's correlation with Benjamini‐Hochberg correction for multiple comparisons was used to relate microbial genera to environmental variables. This analysis revealed strong positive correlations (0.85–0.87) between EC, TDS, and SAL and genera such as *Gimesia*, *Rhodospira*, *Nitrincola*, *Aquarickettsia*, *Pseudodesulfovibrio*, *Sulfurospirillum*, OFTM74, UBA3465, and HK‐STAS‐PATE‐9, indicating a strong influence of salinity‐related factors on these taxa. Most of these genera were exclusively identified in samples from the Delicias lagoon, except for *Sulfurospirillum*, which was also found in Génesis. A distinct pattern was observed for genera such as JAABTL01, JACKEA01, *Pseudofulvimonas*, SKKK01, *Aquiflexum*, *Pontimonas*, and UBA5066, which showed strong positive correlations with pH (0.87–0.90) but no significant correlation with EC, TDS, or SAL. Most of these genera were exclusively identified in the Marvilla lagoon, except for *Pontimonas*, which was only found in Delicias (Figure 8).

## 4 Discussion

### 4.1 Microbial Taxonomic Diversity in the Pantanos de Villa Wetlands

This study represents the first effort to characterize the microbial communities living in these wetlands. Among the unique ASVs identified in all sampled lakes, the majority of bacterial ASVs belonged to the phylum Pseudomonadota, followed by the phylum Bacteroidota. Both phyla are broad categories of bacteria that have adapted to diverse environments. In previous studies on lagoons, these phyla are usually cited as the most abundant (Hou et al. 2024), being widely reported in coastal and urban wetlands, where they fulfill ecological functions such as organic matter degradation, nutrient remineralization, and nitrogen cycle regulation (Wang et al. 2022).

The class Gammaproteobacteria, also abundant in the sampling, has genera involved in denitrification processes and the removal of reduced compounds. Another abundant class, Bacteroidia, has been commonly associated with waterfowl excretions. The elevated presence of these classes suggests an active trophic base, possibly linked to the local abundance of macrofauna such as grebes, herons, and ducks, species observed in these lagoons (Capurro and Díaz 2018). It is important to highlight how the majority of ASVs not identified at the class level belong mostly to these phyla, which would suggest that there are taxa not found in the available databases for this ecosystem.

In the archaeal domain, the unique ASVs belonged mostly to the phylum Nanoarchaeota. This phylum has been associated with reduced‐size archaea, and its presence has been reported in different environments, including aquatic ones (Wurch et al. 2016). When comparing the relative abundance of the identified archaeal classes, the class Nanoarchaeia stands out for its prevalent presence, especially in the Marvilla lagoon, across the three sampling times conducted. Initially, members of the phylum Nanoarchaeota were considered extremophiles. But over time, the incidence of the class Nanoarchaeia has been reported in environments such as marine sediments (Zou et al. 2025). The scope of these taxa remains poorly understood, and our findings indicate that this taxonomic group is also present in coastal wetlands.

Lagoons such as Marvilla or Mayor showed stability in the displayed relative abundance, where different months concurred in their populations despite different sampling months. This phenomenon was not observed in either Delicias or Genesis. This could be due to specific disturbances occurring in these lagoons, which are probably related to human activities, based on the revised literature.

### 4.2 Environmental Factors That Determine the Structure of Microbial Communities in Coastal Wetlands

The Delicias lagoon presented different physicochemical parameters when compared to the other lagoons, specifically in total dissolved solids (TDS), salinity (SAL), conductivity (EC), temperature (TE), and pH. Previous studies have demonstrated that variations in pH and conductivity modify both microbial composition and functionality, affecting processes such as nitrification and phosphate uptake (Chen et al. 2015). A former water quality analysis identified Delicias as the lagoon most affected by anthropogenic contamination, which the study defined as elevated levels of dissolved metals and altered physicochemical parameters such as pH, EC, and DO. This contamination comes from multiple sources, including domestic and industrial wastewater, solid waste, and human activities such as clothes washing (Juárez and Aguirre‐Soto 2024). These conditions in Delicias may exert a selective pressure on the microbial populations of this lagoon, leading to a distinct microbial composition. Specifically, sampling points in Delicias presented a distinct community structure, with a group of microbial genera strongly associated with the anomalous physicochemical parameters of this lagoon.

Another divergent community was the one characterized in the Marvilla lagoon. This lagoon has the characteristic of having the most alkaline pH values of all the sampled lagoons. Taxa such as JACKEA01 stand out in Marvilla, belonging to the Polyangiaceae family, which has a genus known for having the largest genomes at the bacterial level. These have normally been isolated from soil, but there are reports of their presence in aquatic media, specifically in marine waters. They are of biotechnological interest due to their predatory lifestyle, as well as for the compounds they are capable of producing (Garcia and Müller 2014). SKKK01 also stands out, belonging to the family Pirellulaceae, which is characterized by a complex aquatic lifestyle. Most members of this family were isolated from the sea (Kulichevskaya et al. 2022). Marvilla presented the most alkaline pH among all lagoons, which appears to be the main selective factor influencing its microbial populations, as supported by both the dbRDA analysis and Pearson correlation. Additionally, its geographic proximity to the coast may contribute to the divergent structure of its microbial community.

The microbial community in the Mayor lagoon showed the most significant proportion of unclassified ASVs at the class taxonomic rank. This lack of classification can have several reasons. Soriano‐Lerma et al. evaluated the influence of the 16S target region on taxonomic profiles of soil and saliva samples. They conclude that the sequenced 16S region affects the profiling process, and more so in environmental samples (Soriano‐Lerma et al. 2020). Another common bias in these analyses is the used database, where the taxa mostly related to environmental samples have little representation (Furtak et al. 2020). Lastly, experimental design has an influence on possible sequencing errors that might pass some quality filters, and this could affect the taxonomic profiling of the metabarcoding data (Schirmer et al. 2015).

Both the dbRDA and the Pearson's correlation analysis showed that EC, TDS, and SAL display similar association patterns. In the case of dbRDA, the vectors representing these parameters have the same orientation and magnitude. Similarly, in the correlation analysis, the bacterial genera that show a significant association with these three parameters consistently display the same pattern. EC is a measure of the ability of water to conduct electric current, determined by the presence of dissolved salts and inorganic substances that ionize in water. SAL is directly related to electrical conductivity, as it describes the concentration of dissolved salts in water. TDS, on the other hand, is defined as the sum of all particles that can pass through a determined filter, which includes dissolved salts among other materials. These results show this physicochemical relationship in these water samples and suggest that salinity‐related conditions are a major driver of the microbial community composition in these lagoons. The db‐RDA explained 16%–21% of community variation. In microbial ecology this is a common occurrence in the literature, in which substantial variation remains unexplained. Certain authors attribute this to unmeasured environmental factors, biotic interactions, ecological drift or stochastic processes (Zhou and Ning 2017).

These findings can be compared to other studies worldwide. In China, An et al. (2019) determined pH and EC as the most important and influential environmental factors in microbial community structure (An et al. 2019). Likewise, Peralta et al. (2012) described that microbial communities are strongly influenced by pH in the wetlands of the Illinois Department of Transportation in the USA (Peralta et al. 2012). When comparing these results, the coastal wetlands in Pantanos de Villa show a similar pattern of influence by pH and salinity‐related factors (EC, SAL, and TDS).

The sampling season had little influence on microbial community structure, as samples clustered according to sampling site rather than sampling time. However, certain compositional patterns were recognized in lagoons such as Marvilla. The Peruvian coast is characterized by a subtropical desert climate influenced by the Humboldt Current, which results in moderate temperatures with minimal seasonal variation throughout the year (Rundel et al. 1991). Consequently, temperature was not a significant driver of microbial community composition in these wetlands.

Climate change is expected to significantly impact coastal wetlands. Sea level rise, increased frequency of extreme weather events, and altered precipitation patterns are expected to modify the physicochemical conditions of these environments, particularly salinity, pH, and dissolved oxygen levels (Mazhar et al. 2022). Our results show that the composition of microbial communities in the Pantanos de Villa wetlands is driven by salinity‐related variables (EC, TDS, SAL), with certain taxa showing a strong preference for these properties. An alteration of these parameters could lead to a shift in the composition of these communities. Given the ecological importance of microbial communities in nutrient cycling and ecosystem functioning, long‐term monitoring of these wetlands is essential to understand and predict the consequences of climate change on coastal wetland community structure.

The study includes certain caveats. Sample D2.1 exhibited a notably high proportion of reads removed during decontamination (65.82%), substantially higher than other samples (15% on average). This may reflect lower initial DNA concentration or higher susceptibility to contamination. Additionally, sample G3.2 showed unusually high ASV richness compared to other samples; however, this did not appear as an outlier in ordination analyses, suggesting it may represent genuine microbial diversity rather than a technical artifact.

## 5 Conclusions

This study was the first profiling of an ecosystem not previously explored at the microbial level. Based on metabarcoding data information (16S marker), the first approximation of microbial diversity in the Pantanos de Villa wetland was accomplished. The findings in this work describe the influence of anthropogenic contamination on bacteria and archaea. Likewise, they highlight the differences between the communities of each lagoon belonging to the same wetland, where human activities or geographic location exerted selective pressure on them. This opens the possibility for a deeper characterization of the impact of contamination on environmentally relevant ecosystems. Future studies should complement this information with culture‐based approaches and shotgun metagenomic data.

## Author Contributions

**Camila Castillo‐Vilcahuaman:** conceptualization (equal), data curation (equal), formal analysis (lead), investigation (lead), methodology (lead), software (lead), validation (supporting), visualization (lead), writing – original draft (lead), writing – review and editing (lead). **Roger Alberto Palomino Huarcaya:** conceptualization (supporting), writing – original draft (supporting), writing – review and editing (supporting). **Pedro Sepúlveda‐Rebolledo:** methodology (supporting), software (supporting), visualization (supporting), writing – review and editing (supporting). **Maribel Baylon Cortitoma:** conceptualization (equal), data curation (equal), funding acquisition (lead), investigation (equal), methodology (supporting), resources (lead), supervision (lead), writing – original draft (supporting), writing – review and editing (supporting).

## Conflicts of Interest

The authors declare no conflicts of interest.

## Data Availability

The data used in this article is available at GenBank at the following URL: https://www.ncbi.nlm.nih.gov/sra/PRJNA1290511. The code used in this article is available at GitHub at the following URL: https://github.com/reymonera/16S‐lima‐wetlands.
